# Complete Genome Sequence of Serratia marcescens Phage MTx

**DOI:** 10.1128/MRA.00573-19

**Published:** 2019-06-20

**Authors:** Kristin Graham, Miranda Freeman, Heather Newkirk, Mei Liu, Jesse Cahill, Jolene Ramsey

**Affiliations:** a Center for Phage Technology, Texas A&M University, College Station, Texas, USA; Queens College

## Abstract

Serratia marcescens is a nosocomial pathogen that has evolved resistance to multiple antibiotics. Here, we present the genome sequence of myophage MTx that infects S. marcescens. MTx encodes 103 proteins, with 26 being assigned a predicted function or superfamily classification, and it has little similarity with other phages at the nucleotide level.

## ANNOUNCEMENT

Serratia marcescens is a pathogen from the Enterobacteriaceae family responsible for hospital-acquired infections. It is commonly found in soil or water and is able to infect plants, animals, and humans (1). Considering the ability of S. marcescens to form biofilms and its emerging drug resistance, there is a need for new approaches, such as the use of phage, to treat these infections (1, 2). Here, we present the complete genome sequence of the *Serratia*-infecting phage MTx.

Using Serratia marcescens D1 (number 8887172; Ward’s Science), MTx was isolated from activated sludge collected at a wastewater treatment plant located in Bryan, TX. Both the host and phage were cultured as described in reference 3 at 30°C in LB (BD) broth and agar. Phage genomic DNA was purified and prepared with a TruSeq nano low-throughput (LT) kit for Illumina MiSeq sequencing with v2 500-cycle chemistry, which yielded 346,883 reads (4). The 250-bp paired-end reads were quality controlled and trimmed using FastQC (http://www.bioinformatics.babraham.ac.uk/projects/fastqc/) and the FASTX-Toolkit 0.0.14 (hannonlab.cshl.edu/fastx_toolkit/). The MTx contig was assembled using SPAdes v.3.5.0 at 146-fold coverage (5). PCR (forward primer, 5′- GGTTCCTGTGGGTGTAATTGT-3′; reverse primer, 5′- CCTCCTGGCAACACCTTATT-3′) and Sanger sequencing were used to close the genome. Analysis and annotation of the phage genome were performed using tools in Galaxy (https://cpt.tamu.edu/galaxy-pub) and Web Apollo, respectively, both hosted by the Center for Phage Technology at Texas A&M University (6, 7). Gene calling was performed using GLIMMER 3.0 and MetaGeneAnnotator 1.0 or ARAGORN 2.36 for tRNAs, and gene function was predicted using BLASTp v.2.2.31 against the UniProtKB Swiss-Prot/TrEMBL and NCBI nonredundant (nr) databases, with a ≤0.001 expectation value cutoff (8–12). Additionally, domain searches with InterProScan v.5.22, TMHMM v.2.0, and LipoP v.1.0 were used (13–15). Further analysis for predictions used HHpred with ummiclust 30_2018_08 for multiple-sequence alignment (MSA) generation and PDB_mmCIF70 for modeling in the HHsuite v.3.0 release (16). The presence of rho-independent termination sites was detected with TransTermHP v.2.09 (17). Phage samples were stained using 2% (wt/vol) uranyl acetate, and morphology was determined using transmission electron microscopy performed at the Microscopy and Imaging Center at Texas A&M University (18).

MTx has a 68,621-bp genome with a G+C content of 49.9% and a 92.6% coding density from 103 potential protein-coding genes. Predicted functions and superfamily assignments were given to 26 proteins. The genome has no identified tRNAs. PhageTerm predicted 3,566-bp terminal repeats (19). In a comparison using progressiveMauve, MTx does not share significant nucleotide similarity (<30%) with other phages, although it encodes 50 proteins similar to multiple Pseudomonas phages (GenBank accession numbers LC105987, FM897211, LN610579, and KR869157) and 47 proteins similar to Escherichia phage ECML-117 (GenBank accession number JX128258) (20).

The genes in MTx do not appear to be grouped by function, as replication and structural genes are scattered throughout the genome. The enzymatic proteins expected for replication, including DNA polymerase subunits, two helicases, a ligase, and a primase, were annotated. MTx encodes a thymidylate synthase (GenBank accession number QBQ72381). Among the structural components needed for myophage assembly are major and minor capsid proteins and multiple baseplate proteins. MTx contains a lytic transglycosylase (GenBank accession number QBQ72355) and endolysin (GenBank accession number QBQ72363); however, the corresponding holin and spanin components of the typical lysis cassette were not identified.

### Data availability.

The genome sequence and associated data for phage MTx were deposited under GenBank accession number MK618717, BioProject accession number PRJNA222858, SRA accession number SRR8869241, and BioSample accession number SAMN11360401.
